# Social Capital and Oral Health‐Related Quality of Life: A Cross‐Sectional Study Among Periodontitis Patients in Isfahan, Iran

**DOI:** 10.1111/idh.70025

**Published:** 2025-12-04

**Authors:** Omid Fakheran, Parastoo Parhizkar, Fateme Jafari, Mehrnoush Tavakoli, Asieh Maghami‐Mehr

**Affiliations:** ^1^ Division of Oral Surgery and Orthodontics, Department of Dental Medicine and Oral Health Medical University of Graz Graz Austria; ^2^ Dental Students' Research Committee School of Dentistry, Isfahan University of Medical Sciences Isfahan Iran; ^3^ Department of Periodontics, Dental Implants Research Center, Dental Research Institute, School of Dentistry Isfahan University of Medical Sciences Isfahan Iran; ^4^ Department of Statistics Yazd University Yazd Iran

**Keywords:** health‐related quality of life, Oral health, periodontitis, social capital

## Abstract

**Objectives:**

Addressing social determinants of health is one of the essential principles for promoting more equitable oral health outcomes for people. An essential element of the social determinants of health theory is the concept of social and community networks, or social capital. This study aimed to investigate the relationship between social capital and oral health‐related quality of life (OHRQoL) in individuals with periodontal disease.

**Methods:**

In this cross‐sectional study, 300 patients diagnosed with periodontitis (stages II and III) participated. Social capital was assessed using the Onyx and Bullen scale, measuring individuals' available social networks and support. We used the OHIP‐14 questionnaire to assess patients' OHRQoL. A modified version of the Oral Hygiene Self‐Efficacy Questionnaire (OHSQ) was administered as a predictor of oral hygiene behaviour. Additionally, the self‐reported oral health status questionnaire gauged participants' perceptions of their overall oral health. To assess group differences, Mann–Whitney or *T*‐tests were used as part of the analysis. For correlation analysis, we compute Pearson correlation coefficients.

**Results:**

According to the results, the mean score of the social capital scale and quality of life measure was 107.7 ± 22.7 and 5.09 ± 1.23, respectively. There was a positive association between social capital scores and OHRQoL scores with a correlation coefficient of 0.045 among periodontitis patients. However, this association was not statistically significant (*p* value > 0.05). In addition, women's social capital scores were significantly lower than men's score (*p* value < 0.001).

**Conclusion:**

The findings show that among people with periodontitis, social capital scores had a positive correlation with the OHRQoL level.

## 1 Introduction

Historically, biological and behavioural factors have received the most emphasis in studies looking into potential risk factors for periodontal diseases. However, a range of upstream socioeconomic factors, such as poverty, unequal access to health care, lack of education, loneliness, and immigration, were discovered to be significantly linked to a higher prevalence of periodontal disease among adults [1, 2, 3, 4, 5].

Periodontal disease may also be linked to social capital, which has been highlighted as one of the major socioeconomic determinants of oral health [6, 7]. Since the 1990s, the concept of social capital has become widely accepted in the context of public health discourse, opening up a new area of investigation into the social determinants of health [8, 9]. Robert Putnam described social capital as “connections among individuals' social networks and the norms of reciprocity and trustworthiness that arise from them,” and it has been proposed that social capital has an independent relationship with general health [10, 11].

Previous research has also revealed that social capital has a favourable impact on oral health outcomes. The relationship between social capital and dental caries has been examined in some studies. Many studies showed that the higher the level of individual social capital, the less the experiences of tooth decay in children, adolescents and adults [12, 13, 14].

Regarding periodontal disease, some cross‐sectional and longitudinal studies showed that high levels of social capital at the individual and community levels are associated with less gingival bleeding [7, 15, 16]. A few hypothetical explanations for the relationship between periodontal health and social capital have been proposed [17]. Social capital affects periodontal health through a variety of pathways, including behavioural, psychosocial, and health service utilisation [18]. According to the psychosocial pathway, social capital protects periodontal health by reducing the negative effects of stress and fostering resilience, coherence, and a sense of belonging. Social capital may influence periodontal health by encouraging the adoption of healthy behaviours like practicing good oral hygiene and seeking out professional dental care, according to behavioural research and the utilisation of dental service pathways [18].

Nevertheless, there is no available data regarding the association between social capital and perceived consequences of periodontal diseases. Clinical dentistry and research both use a range of outcome measures for evaluating individuals who have periodontitis [19]. However, there could be differences between patient‐perceived well‐being measures and clinical indices such as periodontal attachment loss. Oral health‐related quality of life (OHRQoL) is one such self‐perceived measure that captures the patient's perspective. Additionally, the concept of social capital has gained considerable traction in oral health research in recent years, providing valuable insights for policy makers in many countries.

By considering a wide range of variables, the research aimed to provide a comprehensive understanding of how social capital affects the oral health behaviours and self‐reported oral health status of patients suffering from periodontal disease. Investigating the association between the two can provide valuable insights into how people's social connections and interactions can affect their health behaviours and oral health status. By understanding this relationship, we can identify ways to enhance patients' oral health‐related quality of life through building and fostering social connections.

To our knowledge, no study has been conducted on the role of social capital in the oral health of the population in Iran. Accordingly, this study aimed to investigate the association between oral health‐related quality of life and social capital among periodontal patients in Isfahan, the third largest city in Iran. In addition, the study sought to elucidate the influence of individual and contextual factors on this association.

## 2 Methods and Materials

This study consisted of 300 patients diagnosed with periodontitis (stages II and III). The study received approval from the Ethics Committee of the Isfahan University of Medical Sciences (IR.MUI.RESEARCH.REC.1400.196). We used the STROBE checklist to report this cross‐sectional, observational study [20]. Patient selection was carried out by a board‐certified periodontist at two dental healthcare centers in Isfahan City, Iran. One of these centers was a private dental clinic located in a high‐income neighbourhood. The other one was a public dental healthcare center located in a low‐income neighbourhood. The purpose of choosing these two clinics was to be able to apply maximum variations regarding socio‐economic aspects in our sampling.

The recruitment period for patients spanned from January 2021 to April 2021.

The sample size was determined using Cochran's formula by considering α = 0.05, *d* = 0.1, and a test power of 80% in addition to implementing the OHRQoL and social capital scores as determined in previous studies [21, 22]. Thus an ideal sample size was calculated as *N* = 300. The inclusion criteria for this study were adult patients (≥ 18 years old) with periodontitis (stages II and III) according to the recent periodontal classification, with two or more non‐adjacent interproximal sites showing obvious clinical attachment loss and probing depths more than 3 mm. Patients who were medically compromised, pregnant, or refused to continue with the process were excluded from the study. Until the required number of participants was determined, a convenience sample of patients was recruited based on inclusion and exclusion criteria.

After obtaining written informed consent, patients were requested to complete a demographic questionnaire. The questionnaire includes sections pertaining to age, gender, education, and occupation. To assess the effectiveness of oral hygiene behaviours, specifically brushing and flossing, the modified version of the Oral Hygiene Self‐efficacy Questionnaire (OHSQ) was utilised (Appendix S1). The OHSQ consists of 12 validated items that have been specifically tailored for the Iranian population (Cronbach's alpha = 0.79) (Appendix S2) [23, 24]. These questions were designed to evaluate one's self‐efficacy in maintaining oral hygiene practices across a range of different circumstances. Additionally, we assess the self‐reported oral health of participants by a Likert scale question (Poor—Fair—Good—Very good—Excellent).

The OHIP‐14 questionnaire was administered to assess OHRQoL. The Persian version of the OHIP‐14 questionnaire, with confirmed validity and reliability (Cronbach's alpha = 0.85), was utilised for this assessment [25]. A total of seven subscales were measured in this study, which included functional limitation, physical discomfort, psychological discomfort, physical disability, psychological disability, social disability, and handicap. The scoring range for this questionnaire is from 0 to 56, with higher scores indicating a more unfavourable situation.

Social capital was evaluated using the Onyx and Bullen scale, which consists of multiple items measuring attitudes, openness to diversity, workplace relations, and attitudes towards the government [26]. The Persian version of the Onyx social capital index has been assessed for its validity and reliability, confirming its suitability for use among the Iranian population [27].

To measure social capital, each item was rated on a four‐point Likert scale, ranging from 1 (not at all) to 4 (yes or frequently). Data analysis was conducted using IBM SPSS statistical software version 22.0 (IBM Corp., Armonk, NY, USA). The analysis involved performing Mann–Whitney tests or T‐tests to evaluate group differences. In addition, we calculated Pearson correlation coefficients for correlation analyses. All statistical tests were two‐sided, and *p* values of less than.05 were considered statistically significant.

## 3 Results

Among 300 patients, 128 (42.7%) participants were men and 172 (57.3%) of them were women. The greatest number of participants belonged to the age group of 21–30 years old (33%). More than 75% of patients had at least a high school diploma or higher education, and the majority of them were self‐employed and homemakers (Table 1).

**TABLE 1:** Patients' basic characteristics.

Characteristics		Frequency	Percentage (%)
Age; year	≤ 20 year	15	5
	21–30 year	99	33
	31–40 year	88	29.3
	41–50 year	67	22.3
	≥ 51 year	31	10.3
Sex	Male	128	42.7
	Female	172	57.3
Smoking		21	7
Educational status	Illiterate	2	0.7
	Undergraduate and diploma	164	54.6
	University	134	44.7
Job status	Employee	50	16.7
	Freelancer	123	41
	Homemaker	96	32
	Unemployed	31	10.3

**FIGURE 1:**
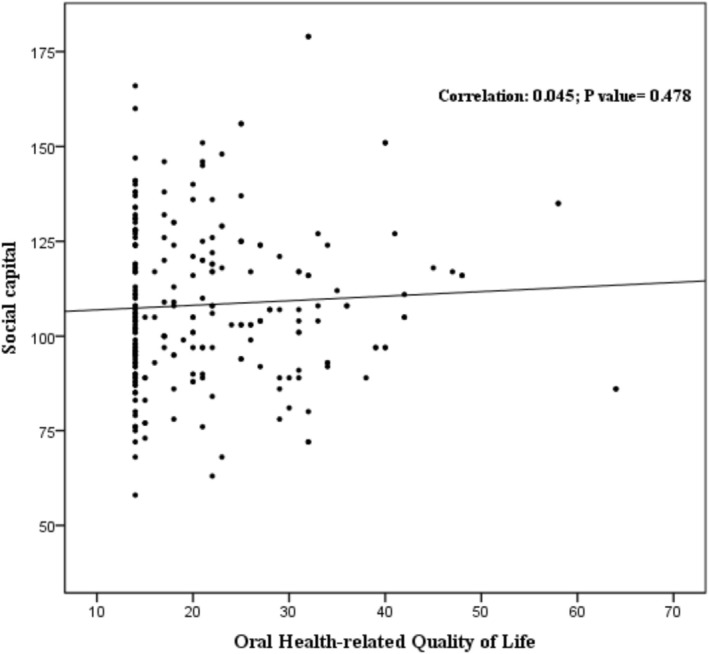
Scatter plot between two variables of social capital and oral health‐related quality of life.

According to the results, the mean score of the social capital scale for women and men were 103/9 ± 22.5 and 115/1 ± 23.4 respectively. The mean score of OHRQoL among the participants was calculated as 5.09 ± 1.23 (Women: 4.15 ± 1.35; Men: 6.68 ± 1.54). There was a positive association between social capital scores and OHRQoL scores with a correlation coefficient of 0.045 among periodontitis patients. However, this association was not statistically significant (Figure 1).

Examining the data regarding oral health behaviours revealed that more than 50% of patients brushed their teeth once a day, while using dental floss was less common among them. In addition, 48.3% of them visited a dentist in the last year. Moreover, 50% of the patients considered their overall oral health to be good, and only 3% of them declared it to be poor. In addition, a large percentage (70.7%) of them had not experienced toothache during the past months (Table 2).

**TABLE 2:** Description of self‐reported oral health status and health behaviours.

Characteristics			Frequency	Percentage (%)
Status of health behaviours	Use of toothbrush	Never	7	2.3
		Less than once a week	11	3.7
		Occasionally	54	18
		Once a day	164	54.7
		Twice a day	52	17.3
		More than twice a day	12	4
	Use of dental floss	Never	97	32.3
		Less than once a week	68	22.7
		Occasionally	58	19.3
		Once a day	58	19.3
		Twice a day	8	2.7
		More than twice a day	11	3.7
Visiting a dentist over the last year			145	48.3%
Status of overall oral health	In your opinion, how is the status of your overall oral health?	Excellent	29	9.7
		Very good	57	19
		Good	150	50
		Fair	55	18.3
		Poor	9	3
	Did you experience toothache in the last month?	Yes	88	29.3
		No	212	70.7

Moreover, women's social capital scores were significantly lower than men's scores. In this regard, the female gender, with a coefficient of 10.42, was associated with lower social capital scores. Furthermore, being employed with a coefficient of 11.07 had a direct and significant role in improving social capital scores (Table 3).

**TABLE 3:** Factors related to participants' social capital and oral health‐related quality of life.

Variables	Beta (95% CI)	S.E	*p*
Social capital			
Sex	−10.42 (−15.68 to −5.16)	2.67	< 0.001
Age	1.96 (−0.52 to 4.46)	1.26	0.121
Smoking	5.99 (−3.32 to 15.30)	4.73	0.206
Education status	1.91 (−3.03 to 6.84)	2.50	0.448
Job status	11.07 (2.97 to 19.16)	4.11	0.008
Oral health‐related quality of life			
Sex	−4.78 (−7.08 to −2.47)	1.17	< 0.001
Age	1.93 (0.85 to 3.02)	0.55	0.001
Smoking	2.82 (−1.33 to 6.98)	2.11	0.182
Education status	3.62 (1.45 to 5.79)	1.10	0.001
Job status	2.47 (−1.25 to 6.19)	1.89	0.192

*Note:* Educational status: non‐university, university education. Employment status: employed, unemployed.

The results also showed that there is a positive correlation between social capital scores and the frequency of patients' tooth brushing, flossing habit and self‐reported oral health status. Nevertheless, these connections lacked statistical significance (Table 4).

**TABLE 4:** Relationship between social capital and oral health‐related quality of life with participant’ health behaviours and self‐reported oral health status.

Variables	Social capital		Oral health‐related quality of life	
	Correlation coefficient	*p*	Correlation coefficient	*p*
Status of health behaviours				
The amount of toothbrush use	0.017	0.779	0.103	0.086
The amount of dental floss use	0.110	0.071	0.053	0.379
Self‐reported oral health status	0.074	0.224	0.050	0.410

In relation to factors associated with oral health‐related quality of life of participants, Gender (women compared to men) had a negative role in OHRQoL score (β = −4.78) while age (β = 1.93) and education level (β = 3.62) had a direct and significant role in this regard (Table 4).

Evaluating the factors related to the patients' oral health behaviours revealed that gender (women compared to men), age, and educational status, with coefficients of 0.57, 0.11, and 0.28, respectively, had a positive role in the subjects' toothbrush frequency (Table 5). Furthermore, the high education level had a significant role in increasing the frequency of dental floss use (β = 0.75). In addition, age and education level with coefficients of 0.08 and 0.15, respectively had a positive and significant role in increasing the patient‐reported oral health status (Table 4).

**TABLE 5:** Factors related to the patients' status of health behaviours and self‐reported oral health status.

Variables	Beta (95% CI)	S.E	*p*
Frequency of tooth brushing			
Sex	0.57 (0.35 to 0.79)	0.11	< 0.001
Age	0.11 (0.01 to 0.21)	0.05	0.045
Smoking	0.37 (−0.04 to 0.77)	0.21	0.077
Education status	0.28 (0.07 to 0.49)	0.10	0.007
Job status	0.11 (−0.24 to 0.45)	0.17	0.539
Frequency of flossing			
Sex	0.17 (−0.16 to 0.51)	0.17	0.313
Age	0.11 (−0.04 to 0.27)	0.08	0.148
Smoking	0.01 (−0.61 to 0.62)	0.31	0.982
Education status	0.75 (0.44 to 1.06)	0.16	< 0.001
Job status	0.26 (−0.58 to 0.46)	0.26	0.835
Overall health			
Sex	−0.01 (−0.13 to 0.12)	0.06	0.930
Age	0.08 (0.02 to 0.13)	0.03	0.010
Smoking	0.05 (−0.18 to 0.27)	0.12	0.695
Education status	0.15 (0.04 to 0.27)	0.06	0.009
Job status	0.06 (−0.13 to 0.26)	0.09	0.536

*Note:* Educational status: non‐university, university education. Employment status: employed, unemployed.

## 4 Discussion

Social capital has become increasingly popular across various research fields in the past few decades. The primary objective of this research was to examine the relationship between social capital and oral health‐related quality of life among individuals diagnosed with periodontal disease. Additionally, the study aimed to explore how both individual factors and contextual aspects influence this association. The results of this study revealed that, despite not being statistically significant, there is a positive relationship between social capital and OHRQoL, as well as social capital and oral hygiene self‐efficacy, among individuals suffering from periodontitis. Furthermore, the level of social capital was negatively associated with gender (i.e., women compared to men). Furthermore, employment was directly and significantly related to higher social capital scores.

The social capital literature has explored various aspects of health, including oral health. It has been mainly discussed in terms of health issues and highlighted as one of the major determinants of oral health outcomes according to the World Health Organization (WHO) [28, 29]. Although there is a broad controversy regarding its definition, previous studies indicated that high levels of social capital are associated with a lower occurrence of chronic diseases, better self‐perception of health, and better quality of life [30, 31].

According to several studies, there is a correlation between having a higher level of social capital and experiencing lower levels of dental caries in individuals of different age groups, including children [12], adolescents [13], and adults [14]. Santiago et al. indicated that communities with higher levels of empowerment have shown a significant decrease in dental caries rates [32]. which was in accordance with the outcome of a 10‐year study, which revealed that a strong social community during early childhood was directly linked to a decrease in dental caries occurrence during adolescence [33].

According to cross‐sectional and longitudinal studies, regarding periodontal conditions, increased social capital is associated with less gingival bleeding at both individual and community levels [7, 15, 16]. Based on the results of the present study, the relationship between social capital and overall oral health was positive which was in agreement with the previously mentioned studies. Moreover, decreased levels of social capital have been linked to increased tooth loss over time [34] and a higher incidence of edentulism [35]. Studying the association between social capital and self‐rated oral health (SROH), regarding subjective outcomes, indicated that high levels of social capital lead to better SROH in children [36], adolescents [37], and adults [34, 35]. Based on the findings of the current study, the relationship between social capital and oral health‐related quality of life (OHRQoL) in patients with periodontitis was direct and it was in line with the previous literature. For instance, Knorst and colleagues assessed the influence of neighbourhood and individual social capital on the OHRQoL of children and indicated that OHRQoL was positively impacted by high levels of social capital within both the individual and neighbourhood contexts during early childhood [38]. According to the findings of Mario V Vettore et al., having stronger social support was associated with improved dental health and better overall OHRQoL. Additionally, having a larger number of social connections was directly correlated with better dental health [37].

In recent decades, there has been a gradual decline in the level of social capital in Iran, with the overall level among the Iranian population being relatively low [39, 40]. Furthermore, recent studies in accordance with our results showed that gender has a notable influence on social capital among citizens in Iran. In a large sample study in Iran, Nedjat et al. reported that women had significantly lower social cohesion scores than men in both the univariate and multivariate analyses [41]. In another cross‐sectional study with 2484 participants, the investigators reported that the mean of the overall social capital was higher among men compared to women in Tehran [40]. The aforementioned findings are consistent with prior research on the decline of traditional social capital and the limited development of modern social capital in Iran [42]. Several studies have indicated that the ongoing urbanisation in Iran has led to a decline in traditional social capital, specifically the bonding type characterised by intergroup affiliations and limited and unique social trust levels [43]. Simultaneously, the development of modern social capital, identified as the bridging type involving intragroup liaisons and widespread trust, has not been fully shaped and established [40, 42]. These results hold significance for forthcoming social development strategies for researchers and policymakers [44].

The mean score of social capital in the current study differs from that found in previous literature. This disparity can be attributed to the absence of a universally accepted method to measure social capital comprehensively [45]. It is worth noting that measuring social capital directly remains a challenge, yet it can be inferred from its determinants or manifestations. Consequently, one could argue that understanding social capital requires an examination of its underlying factors and observable outcomes [45, 46]. Similarly, the mean scores of OHRQoL varied significantly across diverse cultures. These cultural nuances play a significant role in shaping individuals' perceptions and experiences related to their oral health, ultimately influencing their overall quality of life. By considering the diverse cultural backgrounds and beliefs of individuals, we can gain a deeper understanding of how these differences may contribute to variations in mean scores [47].

This study has some limitations. First, because our study was cross‐sectional, we were unable to identify the causative pathways. There could be a reverse causality issue: some patients with lower oral health status might have fewer interactions with the community for psychosocial reasons. Second, according to our recruitment protocol, we only screened individuals to identify patients who met the inclusion criteria. Therefore, in this study, we did not include clinical parameters such as DMFT or periodontal pocket depth in the statistical analysis. Providing clinical data in future studies may help improve our knowledge in this regard. Third, participants were chosen from just two dental clinics in a sizable Iranian metropolis. Consequently, selection bias might have had an impact on the outcomes. Therefore, it is not apparent whether our findings apply to other areas or communities. As such, multistage sampling strategies might be used to cover a larger number of people in this geographic region in future studies attempting to expand knowledge in this respect.

Additional investigation is necessary to gain a deeper understanding of how social capital can influence oral health outcomes. The future focus and challenges in this field predominantly revolve around creating interventions and implementing health promotion initiatives such as “Social Prescribing” to address social determinants of oral health. Social prescribing is an effort to apply the common knowledge that people's health is largely determined by socioeconomic factors, and that people who have access to social supports within their communities are healthier [48, 49]. These factors are beyond the service scope of healthcare professionals, but account for more than half of the determinants of health and wellbeing [50].

## 5 Conclusion

The results of this study suggested that there is a positive relationship between social capital and oral health‐related quality of life among individuals suffering from periodontitis. Future studies will be able to provide further insight regarding this association, particularly concerning how social capital operates among other upstream social determinants of oral health.

## 6 Clinical Relevance

### 6.1 Scientific Rationale for Study

Social capital may have an impact on improving oral health among dental patients. However, no study has explored the association between social capital and self‐perceived oral health measures. This study explores the association between social capital and OHRQoL in individuals with periodontal disease.

### 6.2 Principal Findings

Among the periodontal patients participating in this study, individuals who have less social support are more likely to have a lower oral health‐related quality of life.

### 6.3 Practical Implications

When caring for periodontitis patients, it can be an advantage to consider a holistic care approach that not only takes into account the physical needs of patients but also considers their psychosocial needs.

## Author Contributions

P.P. had full access to the data, carried out the data analysis and drafted the manuscript. F.J. participated in the design of the study, carried out the survey and participated in the data analysis. M.T. participated in the design of the study, data analysis and interpretation and review of the paper. A.M.‐M. participated in data analysis and the interpretation of the data. O.F. participated in the study design, data analysis and interpretation, drafting and review of the manuscript, and obtained funding. All authors read and approved the final manuscript.

## Funding

This research was funded by Isfahan University of Medical Sciences (IR.MUI.RESEARCH.REC.1400.196).

## Conflicts of Interest

The authors declare no conflicts of interest.

## Supporting information


**Appendix S1:** Supporting information.

**Appendix S2:** Supporting information.

## Data Availability

The data that support the findings of this study are available on request from the corresponding author. The data are not publicly available due to privacy or ethical restrictions.
