# Supplementary material for: Social Capital and Oral Health‐Related Quality of Life: A Cross‐Sectional Study Among Periodontitis Patients in Isfahan, Iran
Source: Int J Dent Hyg. 2025 Dec 4;24(1):100–7. doi: 10.1111/idh.70025 (PMC12748048; doi:10.1111/idh.70025)
Supplement: Supplementary file 1 — Appendix S1: Supporting information. [file IDH-24-100-s002.docx]

ONLINE Appendix S1

**Modified oral hygiene-related self-efficacy questionnaire (OHSE). Questions could be answered with “Completely confident not to” (1), “Fairly confident not to” (2), “Fairly confident to” (3), “Completely confident” (4) with the possible points in parentheses.**

| **Tooth brushing self-efficacy**  **How confident are you, that you brush your teeth in the following situations?**  When you are tired in the evening  When you are not going to a dentist in near future  When you are on holiday  When you have a lot of work  When you have a headache  When you feel ill |
| --- |
| **Inter-dental cleaning self-efficacy**  **How confident are you, that you clean your proximal surfaces in the following situations?**  When you are tired in the evening  When you are not going to a dentist in near future  When you are on holiday  When you have a lot of work  When you have a headache  When you feel ill |
