# Supplementary material for: Social Capital and Oral Health‐Related Quality of Life: A Cross‐Sectional Study Among Periodontitis Patients in Isfahan, Iran
Source: Int J Dent Hyg. 2025 Dec 4;24(1):100–7. doi: 10.1111/idh.70025 (PMC12748048; doi:10.1111/idh.70025)
Supplement: Supplementary file 2 — Appendix S2: Supporting information. [file IDH-24-100-s001.pdf]

ONLINE Appendix S2

Persian version of Modified oral hygiene-related self-efficacy questionnaire (OHSE)

پرسشنامه خودکارآمدی بهداشت دهان و دندان. این پرسشنامه شامل گویه هایی مرتبط با رفتارهای بهداشتی دهان و دندان بود که نسبت به موقعیت های مختلف نمره ای به فرد اختصاص میداد. این پرسش ها میتوانست با گزینه های "کاملا مطمئنم انجام نمیدهم (1)، تقریبا مطمئنم انجام نمیدهم (2)، تقریبا مطمئنم انجام میدهم (3)، کاملا مطمئنم انجام میدهم (4)" پاسخ داده شود.

**خودکارآمدی مسواک**<br>**چقدر مطمئن هستید که در شرایط زیر دندان های خود را مسواک می زنید؟**<br><br>شب هایی که خسته هستید<br>وقتی در آینده ای نزدیک قصد رفتن به دندانپزشکی را دارید<br>وقتی در تعطیلات و یا مسافرت هستید<br>وقتی کارهای زیادی برای انجام دادن دارید<br>وقتی دجار سردرد شده اید<br>وقتی احساس ناخوشی میکنید
**خودکارآمدی نخ دندان**<br>**چقدر مطمئن هستید که در شرایط زیر بین دندان های خود را (با نخ دندان) تمیز میکنید؟**<br><br>شب هایی که خسته هستید<br>وقتی در آینده ای نزدیک قصد رفتن به دندانپزشکی را دارید<br>وقتی در تعطیلات و یا مسافرت هستید<br>وقتی کارهای زیادی برای انجام دادن دارید<br>وقتی دجار سردرد شده اید<br>وقتی احساس ناخوشی میکنید
